# 430. Clinical Features and Outcomes of COVID-19 Hospitalized Adults by Variant

**DOI:** 10.1093/ofid/ofad500.500

**Published:** 2023-11-27

**Authors:** Maria A Perez, Ashley Tippett, Ludy R Carmola, Laila Hussaini, Luis W Salazar, Olivia Reese, Caroline R Ciric, Chris Choi, Elizabeth G Taylor, Wensheng Li, Hui-Mien Hsiao, Kathy Stephens, Theda Gibson, Laura A Puzniak, Robin Hubler, Timothy L Wiemken, Srinivas Valluri, Benjamin Lopman, Satoshi Kamidani, Evan J Anderson, Christina A Rostad, Jesse J Wagonner, Anne Piantadosi

**Affiliations:** Emory University, Atlanta, Georgia; Emory University, Atlanta, Georgia; Emory University School of Medicine, Atlanta, Georgia; Emory Univeristy, Atlanta, Georgia; Emory University, Atlanta, Georgia; Emory University, Atlanta, Georgia; Emory University, Atlanta, Georgia; Emory University, Atlanta, Georgia; Emory University School of Medicine, Atlanta, Georgia; Emory University School of Medicine, Atlanta, Georgia; Emory University School of Medicine, Atlanta, Georgia; Emory University School of Medicine, Atlanta, Georgia; Emory University School of Medicine, Atlanta, Georgia; Pfizer Inc., Collegeville, Pennsylvania; Pfizer Inc., Collegeville, Pennsylvania; Pfizer Inc, Saint Louis, Missouri; Pfizer Inc, Saint Louis, Missouri; Rollins School of Public Health | Emory University, Atlanta, Georgia; Emory University School of Medicine and Children's Healthcare of Atlanta, Atlanta, Georgia; Emory University School of Medicine, Atlanta, Georgia; Emory University School of Medicine and Children's Healthcare of Atlanta, Atlanta, Georgia; Emory University, Atlanta, Georgia; Emory University School of Medicine, Atlanta, Georgia

## Abstract

**Background:**

SARS-CoV-2 has changed and mutated over time. It is important to evaluate changes in clinical presentation and outcomes based on the emerging variants. In this study, we aimed to compare differences in symptoms and outcomes among adults hospitalized with COVID-19 by variant.

**Methods:**

From May 2021 to August 2022, we enrolled adults ≥ 18 years of age hospitalized with acute respiratory infection (ARI) at two Emory University hospitals. Demographic and clinical information were obtained from participant interviews and medical chart abstractions. Enrolled patients provided nasopharyngeal and oropharyngeal swabs and standard-of-care specimens. Samples which tested positive for SARS-CoV-2 by molecular testing were subjected to SARS-CoV-2 targeted spike SNP PCR and viral genome sequencing to determine a variant. Statistical analysis was performed using SAS version 9.4. Bivariable analyses were conducted to compare characteristics and identify independent characteristics associated with each variant.

**Figure:**
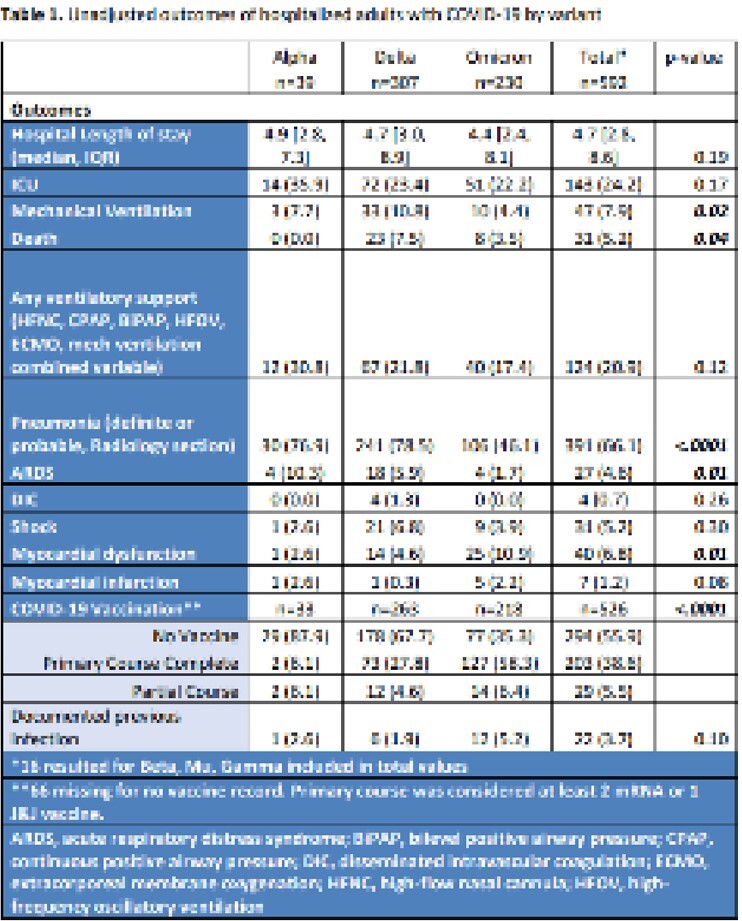

**Results:**

Of 1677 ARI enrolled participants, 850 tested positive for SARS-CoV-2, of whom 592 had a variant identified by either SNP PCR or full genome sequencing. The distribution of variants among these cases were as follows: 39 Alpha (6.6%), 2 Beta (0.3%), 307 Delta (51.9%), 9 Gamma (1.5%), 5 Mu (0.8%), and 230 Omicron (38.9%). When analysis was limited to participants with Alpha, Delta, or Omicron, those with Omicron were significantly older, white, female, and had underlying comorbidities. Compared to participants with Alpha and Delta, those with Omicron more often had sore throat and abdominal pain, but less often had fever, diarrhea, anosmia, ageusia, or shortness of breath. Also, those with Omicron were more often partially or fully vaccinated, with the majority of Omicron infections occurring after vaccination (Table 1). Most clinical outcomes were better among those with Omicron infections, while participants with Delta had the highest proportion of radiographic pneumonia, mechanical ventilation, and death (Table 1).

**Conclusion:**

SARS-CoV-2 variants were associated with distinct clinical characteristics and outcomes, and the Delta variant was associated with the highest frequency of pneumonia, mechanical ventilation, and death.

**Disclosures:**

**Laura A. Puzniak, PhD. MPH**, Pfizer, Inc.: Employee|Pfizer, Inc.: Stocks/Bonds **Robin Hubler, MS**, Pfizer, Inc.: Employee|Pfizer, Inc.: Stocks/Bonds **Timothy L. Wiemken, PhD**, Pfizer Inc: Employee|Pfizer Inc: Stocks/Bonds **Srinivas Valluri, PhD**, Pfizer Inc: Pfizer Employee and hold Pfizer stocks/options|Pfizer Inc: Ownership Interest|Pfizer Inc: Stocks/Bonds **Benjamin Lopman, PhD**, Epidemiological Research and Methods, LLC: Advisor/Consultant|Hillevax, Inc: Advisor/Consultant **Satoshi Kamidani, MD**, CDC: Grant/Research Support|Emergent BioSolutions: Grant/Research Support|NIH: Grant/Research Support|Pfizer Inc: Grant/Research Support **Evan J. Anderson, MD**, GSK: Advisor/Consultant|GSK: Grant/Research Support|Janssen: Advisor/Consultant|Janssen: Grant/Research Support|Kentucky Bioprocessing, Inc.: Safety Monitoring Board|Moderna: Advisor/Consultant|Moderna: Grant/Research Support|Moderna: Currently an employee|Moderna: Stocks/Bonds|Pfizer: Advisor/Consultant|Pfizer: Grant/Research Support|Sanofi Pasteur: Advisor/Consultant|Sanofi Pasteur: Grant/Research Support|Sanofi Pasteur: Safety Monitoring Board|WCG/ACI Clinical: Data Adjudication Board **Christina A. Rostad, MD**, BioFire Inc.: Grant/Research Support|GlaxoSmithKline Biologicals: Grant/Research Support|Janssen: Grant/Research Support|MedImmune LLC: Grant/Research Support|Meissa Vaccines, Inc.: RSV vaccine technology|Merck & Co., Inc.: Grant/Research Support|Micron Technology, Inc.: Grant/Research Support|Moderna, Inc.: Grant/Research Support|Novavax: Grant/Research Support|PaxVax: Grant/Research Support|Pfizer, Inc.: Grant/Research Support|Regeneron: Grant/Research Support|Sanofi Pasteur: Grant/Research Support

